# Delivery of Cancer Care in Ontario, Canada, During the First Year of the COVID-19 Pandemic

**DOI:** 10.1001/jamanetworkopen.2022.8855

**Published:** 2022-04-25

**Authors:** Meghan J. Walker, Jonathan Wang, Joshua Mazuryk, Siew-Mei Skinner, Olivia Meggetto, Eta Ashu, Steven Habbous, Narges Nazeri Rad, Gabriela Espino-Hernández, Ryan Wood, Munaza Chaudhry, Saba Vahid, Julia Gao, Daniela Gallo-Hershberg, Eric Gutierrez, Claudia Zanchetta, Deanna Langer, Victoria Zwicker, Michelle Rey, Martin C. Tammemägi, Jill Tinmouth, Rachel Kupets, Anna M. Chiarelli, Simron Singh, Padraig Warde, Leta Forbes, Julian Dobranowski, Jonathan Irish, Linda Rabeneck

**Affiliations:** 1 Ontario Health–Cancer Care Ontario, Toronto, Canada; 2 Dalla Lana School of Public Health, University of Toronto, Toronto, Canada; 3 Leslie Dan Faculty of Pharmacy, University of Toronto, Toronto, Canada; 4 Department of Health Sciences, Brock University, St Catharines, Canada; 5 ICES, Toronto, Canada; 6 Department of Medicine, Sunnybrook Health Sciences Centre, Toronto, Canada; 7 Department of Medicine, University of Toronto, Toronto, Canada; 8 Odette Cancer Centre, Sunnybrook Health Sciences Centre, Toronto, Canada; 9 Department of Obstetrics and Gynecology, University of Toronto, Toronto, Canada; 10 Department of Medical Oncology, Sunnybrook Health Sciences Centre, Toronto, , Canada; 11 Radiation Medicine Program, Princess Margaret Cancer Centre, Toronto, Canada; 12 Department of Radiation Oncology, University of Toronto, Toronto, Canada; 13 Department of Medical Oncology, R.S. McLaughlin Durham Regional Cancer Centre, Oshawa, Canada; 14 Department of Radiology, McMaster University, Hamilton, Canada; 15 Niagara Health, St Catharines, Ontario, Canada; 16 Department of Otolaryngology–Head and Neck Surgery, University of Toronto, Toronto, Canada; 17 Department of Surgical Oncology, Princess Margaret Cancer Centre, Toronto, Canada

## Abstract

**Importance:**

The COVID-19 pandemic has impacted cancer systems worldwide. Quantifying the changes is critical to informing the delivery of care while the pandemic continues, as well as for system recovery and future pandemic planning.

**Objective:**

To quantify change in the delivery of cancer services across the continuum of care during the COVID-19 pandemic.

**Design, Setting, and Participants:**

This population-based cohort study assessed cancer screening, imaging, diagnostic, treatment, and psychosocial oncological care services delivered in pediatric and adult populations in Ontario, Canada (population 14.7 million), from April 1, 2019, to March 1, 2021. Data were analyzed from May 1 to July 31, 2021.

**Exposures:**

COVID-19 pandemic.

**Main Outcomes and Measures:**

Cancer service volumes from the first year of the COVID-19 pandemic, defined as April 1, 2020, to March 31, 2021, were compared with volumes during a prepandemic period of April 1, 2019, to March 31, 2020.

**Results:**

During the first year of the pandemic, there were a total of 4 476 693 cancer care services, compared with 5 644 105 services in the year prior, a difference of 20.7% fewer services of cancer care, representing a potential backlog of 1 167 412 cancer services. While there were less pronounced changes in systemic treatments, emergency and urgent imaging examinations (eg, 1.9% more parenteral systemic treatments) and surgical procedures (eg, 65% more urgent surgical procedures), major reductions were observed for most services beginning in March 2020. Compared with the year prior, during the first pandemic year, cancer screenings were reduced by 42.4% (−1 016 181 screening tests), cancer treatment surgical procedures by 14.1% (−8020 procedures), and radiation treatment visits by 21.0% (−141 629 visits). Biopsies to confirm cancer decreased by up to 41.2% and surgical cancer resections by up to 27.8% during the first pandemic wave. New consultation volumes also decreased, such as for systemic treatment (−8.2%) and radiation treatment (−9.3%). The use of virtual cancer care increased for systemic treatment and radiation treatment and psychosocial oncological care visits, increasing from 0% to 20% of total new or follow-up visits prior to the pandemic up to 78% of total visits in the first pandemic year.

**Conclusions and Relevance:**

In this population-based cohort study in Ontario, Canada, large reductions in cancer service volumes were observed. While most services recovered to prepandemic levels at the end of the first pandemic year, a substantial care deficit likely accrued. The anticipated downstream morbidity and mortality associated with this deficit underscore the urgent need to address the backlog and recover cancer care and warrant further study.

## Key Points

**Question:**

Did the delivery of services within a cancer system change during the first year of the COVID-19 pandemic?

**Findings:**

This population-based cohort study conducted in Ontario, Canada, found a total of 4 476 693 cancer care services during the first year of the COVID-19 pandemic, compared with 5 644 105 services in the year prior, representing a reduction of 20.7% and suggesting a backlog of 1 167 412 cancer services during the first pandemic year. Limited change was observed in systemic treatments and emergency or urgent imaging examinations and surgical procedures, while major reductions were observed in cancer screening tests, biopsies, surgical treatments, and new consultations for systemic and radiation treatment.

**Meaning:**

These findings provide evidence on the deficits in cancer care that occurred during the first year of the COVID-19 pandemic that are likely to inform continued delivery of care, recovery, and future pandemic planning.

## Teaser

This cohort study assesses changes in the volume of cancer care episodes during the first year of the COVID-19 pandemic in Ontario, Canada.

## Introduction

On March 11, 2020, a global pandemic of COVID-19 was declared by the World Health Organization.^1^ To mitigate COVID-19 transmission and preserve health system capacity, the provincial government of Ontario, Canada, directed nonemergent health care to cease across the province.^2^ Cancer screening was suspended on March 23, 2020, and some cancer diagnostic procedures and treatments were deferred. Cancer treatment protocols were adapted to offset risk of surgical delay (eg, neoadjuvant vs adjuvant chemotherapy) or reduce the risk of COVID-19 exposure in patients who were immunocompromised by eliminating some hospital visits (eg, switch from parenteral to oral systemic therapy or radiation hypofractionation treatment approaches). Deferred services were permitted to resume gradually in Ontario beginning on May 26, 2020.

Changes to the delivery of cancer care have been reported worldwide.^3,4,5,6,7,8,9^ There is an abundant literature describing changes to individual cancer services; however, to our knowledge, this is the first population-based study to describe changes to a broad range of services within a single jurisdiction-wide cancer system. This study generates knowledge regarding the association between disruptions in upstream services and downstream services as well as the magnitude of the backlog that accumulated across a system during the first pandemic year. This information is critical to informing delivery of care while the pandemic continues to exert pressure on health systems, coordinated approaches to cancer service recovery, preparation for changes in cancer outcomes, and future pandemic planning.

## Methods

This cohort study adheres to privacy regulations, ethics review and informed consent was not required. Ontario Health–Cancer Care Ontario is designated a prescribed entity for the purposes of section 45(1) of the Personal Health Information Protection Act of 2004. As a prescribed entity, Ontario Health–Cancer Care Ontario is authorized to collect personal health information from health information custodians without the consent of the patient and to use such personal health information for the purpose of analysis or compiling statistical information with respect to the management, evaluation or monitoring of the allocation of resources to or planning for all or part of the health system, including the delivery of services. This study is reported following the Strengthening the Reporting of Observational Studies in Epidemiology (STROBE) reporting guideline.

### Study Design and Population

Health services are delivered to Ontario’s population of 14.7 million through a publicly funded single-payer provincial health care system. The province is subdivided into administrative health regions (West, Central, Toronto, East, and North), and service delivery is overseen by Ontario Health, a provincial government agency. Cancer services are overseen by Ontario Health–Cancer Care Ontario and delivered through 14 Regional Cancer Programs (networks of hospitals and other health care organizations). Approximately 85 000 new cancer cases are diagnosed in Ontario annually.

Following the pandemic declaration, Ontario Health–Cancer Care Ontario activated its crisis management protocol. As part of this activity, pandemic clinical guidance documents were rapidly developed, informed by guidance developed in 2009 during the *H1N1* pandemic and recent evidence.^10,11^ An evaluation of the impact of the pandemic on the Ontario cancer system is under way to support system response and recovery. The first phase, reported here, encompassed a cohort study quantifying change in the delivery of care in 7 areas, including screening, imaging, surgical treatment, pathological reporting, systemic treatment, radiation treatment, and psychosocial oncological care. Individuals of any age who received 1 or more cancer service in Ontario from January 1, 2019, to March 31, 2021, were eligible for inclusion.

### Measures

#### Cancer Screening

Ontario has 4 organized cancer screening programs: the Ontario Breast Screening Program, which recommends mammography for people at average risk, and mammography plus breast magnetic resonance imaging (MRI) for people at high risk; the Ontario Cervical Screening Program, which recommends cervical cytological examination for people with a cervix; ColonCancerCheck, which recommends the fecal immunochemical test for people at average risk and colonoscopy for people at increased risk; and the Ontario Lung Screening Program (OLSP), which screens people at high risk with low-dose computed tomography (LDCT) of the chest. Monthly cancer screening test and diagnostic procedure (eg, colonoscopy, colposcopy) volumes from January 1, 2019, to March 31, 2021, were included.

#### Cancer Imaging 

Medical imaging is performed in hospital and nonhospital settings in Ontario. CT and MRI are categorized by priority level with corresponding target wait times, including priority 1 (P1; emergent, 24-hour target), priority 2 (P2; urgent, 48-hour target), priority 3 (P3; semiurgent, 10-day target), and priority 4 (P4; nonurgent, 28-day target). Our analysis focused on MRI and CT owing to preexisting pressures on these resources, their critical role in cancer care, and data availability. Positron emission tomography (PET) scan volumes were included owing to the role of PET in oncology care. Monthly volumes of P2 to P4 adult and pediatric MRI and CT scans for cancer diagnosis and staging, and PET scans with oncology indications from January 1, 2019, to March 31, 2021, were included. P1 scans were excluded, since they were less acutely affected during the pandemic. Less than 1% of PET scans were performed for the nononcology indication of epilepsy.

#### Pathological Reporting

Cancer pathological reports are received by the Ontario Cancer Registry in near real-time and serve as the initial identification of a patient within the cancer system for most cases. Pathological reports were used as a proxy measure for cancer diagnoses. Weekly biopsy and resection report volumes from December 31, 2018, to April 4, 2021, were included. Only reports of tumors with malignant behavior were included (*International Classification of Diseases for Oncology, Third Edition* [*ICD-O-3*] behavior code 3).

#### Cancer Treatment Surgical Procedures

In Ontario, surgical treatments are also categorized by priority to standardize wait times. P1 procedures are those considered “life or limb” emergencies (ie, 24-hour maximum wait time target), while P2 to P4 procedures have varying maximum wait time targets (P2, 14 days; P3, 28 days; and P4, 84 days). Monthly P2 to P4 adult cancer treatment surgical procedure volumes from January 1, 2019, to March 31, 2021, were included. P1 procedures were excluded, since they were less acutely affected during the pandemic. Pediatric surgical cancer treatments were excluded because they are combined with all other pediatric surgical procedures.

#### Systemic Therapy

Monthly adult systemic treatment visit volumes from January 1, 2019, to March 31, 2021, were included. Visit types included new case consultations, total systemic suite visits (nonoral antineoplastic agents, supportive agents, transfusions, hydration therapy, or came for treatment but were too ill to be treated), and follow-up visits. Systemic treatment visits were examined by type, including antineoplastic parenteral, antineoplastic oral, or supportive or adjunctive therapy (eg, parenteral bisphosphonates, hydration therapy).

#### Radiation Therapy

Monthly adult and pediatric radiation therapy visit volumes for new patient consultations, radiation treatment visits, and follow-up visits from January 1, 2019, to March 31, 2021, were included. Radiation therapy visits were examined by intent.

#### Psychosocial Oncological Care

Many types of psychosocial oncological care interventions are available in Ontario, including individual, family, and group counseling or psychotherapy; practical support; lymphedema clinics; rehabilitation services; nutritional support; and support with speech and swallowing issues. Monthly new and follow-up psychosocial oncological care visit volumes from January 1, 2019, to March 31, 2021, were included.

### Data Sources

Data were extracted from 9 provincial health administrative databases (eTable in Supplement 1). LDCT and PET scan data were extracted from hospital records submitted to Ontario Health.

### Statistical Analysis

For cancer screening tests, diagnostic colonoscopies and colposcopies, imaging, cancer treatment, and psychosocial oncological care services, the difference between monthly volumes performed in 2020 and 2021 and the corresponding month of 2019 were calculated, reporting absolute volume and percentage changes. Absolute volume and percentage changes were calculated comparing the first year of the pandemic (April 1, 2020-March 31, 2021) to the previous year (April 1, 2019-March 31, 2020) and used to estimate backlog volumes, as well as percentage change at the 6-month mark. For pathological reports, the volume of resection and biopsy reports from each week in 2019, 2020, and 2021 were plotted, and a pre–COVID-19 mean (54 weeks prior to the pandemic) and post–COVID-19 mean (54 weeks following the pandemic declaration) were calculated. The volume and percentage of systemic and radiation therapy, and psychosocial oncological care visits conducted virtually (telephone, video conference) vs in-person were calculated.

Data were assessed using Excel (Microsoft). Data were analyzed from May 1 to July 31 2021.

## Results

In the first year of the pandemic, there were a total of 4 476 693 cancer care services, compared with 5 644 105 services in the year prior, a difference of 20.7% fewer services compared with the previous year, representing a potential backlog of 1 167 412 cancer services (Table). Cancer screening tests were reduced by 42.4% (from 2 395 169 to 1 378 988 screening tests), a difference of 1 016 181 tests, accounting for 87.0% of the total backlog. Other high-volume changes included diagnostic or surveillance colonoscopy (difference: −62 775 colonoscopies [−18.8%]), colposcopy (difference: −21 013 colonoscopies [−20.6%]), cancer treatment surgical procedures (−8020 procedures [−14.1%]), and radiation treatments (−141 629 treatments [−21.0%]).

**Table.** Cancer Service Volumes in Ontario, Canada, Pre–COVID-19 Pandemic vs During the COVID-19 Pandemic

Service	Pre–COVID-19	During COVID-19	Backlog volume^a^	Change, %	
				At 6 mo^b^	Cumulative^c^
Cancer screening tests					
All	2 395 169	1 378 988	−1 016 181	−66.4	−42.4
Fecal test (GFOBT or FIT)	672 406	346 878	−325 528	−77.8	−48.4
Screening colonoscopy^d^	120 719	66 404	−54 315	−65.6	−45.0
Cervical cytology test	892 616	551 222	−341 394	−59.0	−38.3
Mammogram^e^	691 978	397 126	−294 852	−67.5	−42.6
Breast MRI^e^	11 664	11 812	+148	−29.0	+1.3
Thoracic LDCT^f^	5786	5546	−240	−45.8	−4.1
Diagnostic assessment procedures					
All	435 861	352 073	−83 788	−38.3	−19.2
Colonoscopy^g^	333 965	271 190	−62 775	−39.7	−18.8
Colposcopy	101 896	80 883	−21 013	−33.3	−20.6
Cancer imaging examinations					
All	400 178	409 011	+8833	−2.1	+2.2
MRI (staging and diagnosis)	70 926	70 945	+19	−6.3	+0.03
CT (staging and diagnosis)	309 148	316 419	+7271	−1.5	+2.3
PET	20 104	21 647	+1543	+4.2	+7.7
Cancer treatment visits					
All	2 316 792	2 233 256	−83 536	−7.3	−3.6
Cervical precancer treatment	8572	6856	−1716	−28.3	−20.0
Cancer treatment surgical procedure, priority 2-4^h^	56 735	48 715	−8020	−21.1	−14.1
Systemic therapy					
New consultation	72 459	66 959	−5500	−15.1	−8.2
Follow-up visit	644 496	710 642	+66 146	+7.1	+9.3
Suite visits (total)^i^	483 577	466 555	−17 022	−4.5	−3.5
Parenteral visit	390 450	398 013	+7563	+1.8	+1.9
Supportive or adjunctive treatment visit	83 992	60 941	−23 051	−31.1	−27.4
Oral antineoplastic treatment visit	141 749	141 447	−302	−0.1	−0.2
Radiation treatment					
New consultation	51 669	46 886	−4783	−14.8	−9.3
Follow-up visit	182 700	211 990	+29 290	+11.3	+16.0
Visit	674 835	533 206	−141 629	−22.8	−21.0
Psychosocial oncological care					
All	96 105	103 365	+7260	+5.2	+7.5
New visit	24 873	25 183	+310	−1.5	+1.2
Follow-up visit	71 232	78 182	+6950	+7.6	+9.8
Total	5 644 105	4 476 693	1 167 412	−31.1	−20.7

Abbreviations: FIT, fecal immunochemical test; GFOBT, guaiac fecal occult blood test; LDCT, low-dose computed tomography; MRI, magnetic resonance imaging; PET, positron emission tomography.

^a^ Estimated as the difference between expected (based on observed volume in April 2019–March 2020) and observed volumes (April 2020–March 2021).

^b^ Percentage change in volume for the first 6 months of the pandemic (April to October 2020) vs the same period the previous year (April to October 2019).

^c^ Percentage change in volume for the first year of the pandemic (April 2020 to March 2021) vs the same period the previous year (April 2019 to March 2020).

^d^ Includes screening colonoscopies performed in individuals with a family history of colorectal cancer or other risk factors.

^e^ Includes mammograms and breast MRIs performed through the Ontario Breast Screening Program.

^f^ Includes LDCT scans performed through the Ontario Lung Screening Program.

^g^ Includes colonoscopies performed for follow-up of abnormal fecal test result, symptomatic, or surveillance.

^h^ Includes all oncological surgical procedures, including lymphoma and skin (melanoma, carcinoma) procedures. Emergency procedures were excluded.

^i^ Includes patients who received nonoral antineoplastic agents, came for antineoplastic treatment but were too ill to be treated, received only supportive agents, or received transfusions or hydration therapy.

### Cancer Screening

**Figure 1.**
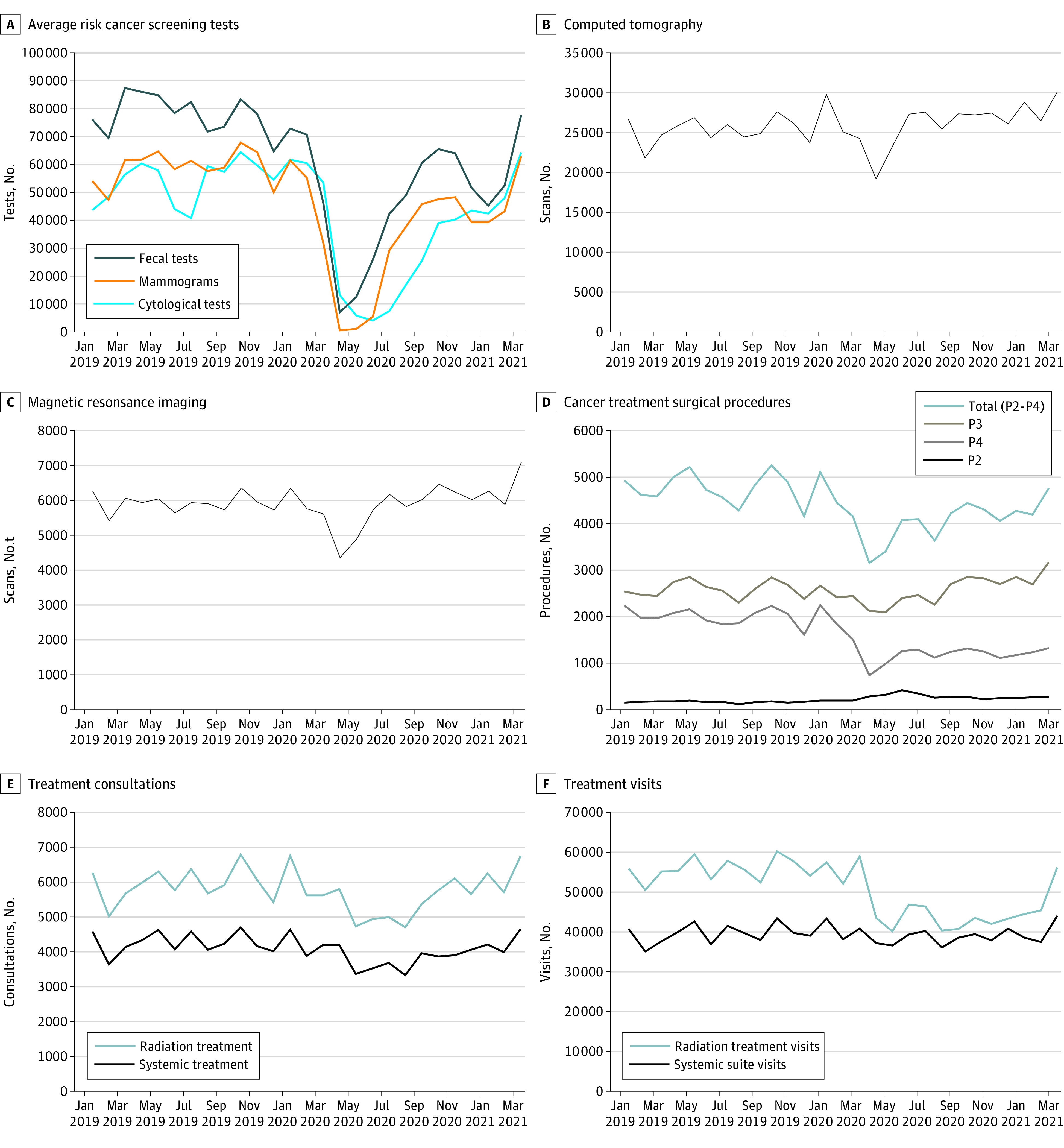
Cancer Service Volumes, Ontario, January 1, 2019, to March 31, 2021 P2 indicates urgent treatment with a 48-hour target; P3, semiurgent treatment with a 10-day target; and P4, nonurgent treatment with a 28-day target.

Reductions in screening tests recommended for individuals at average risk (ie, fecal tests, mammograms, cervical cytological tests) were greatest in April to June 2020 (range, −67.5% to −99.8%), corresponding to the suspension of nonemergent health services (Figure 1). Cancer screening test volumes remained below prepandemic levels through March 2021 (Figure 1), except for high-risk breast and lung screening (eFigure 1 in Supplement 1).

### Cancer Imaging

Volumes decreased in April and May 2020 for CT (−25.9% and −13.3%) and MRI (−26.7% and −19.3%), recovered to prepandemic levels by June 2020, and exceeded 2019 levels in 2021 (Figure 1). PET scan volumes were similar to or exceeded prepandemic volumes in the first year (eFigure 2 in Supplement 1).

### Pathological Reporting

**Figure 2.**
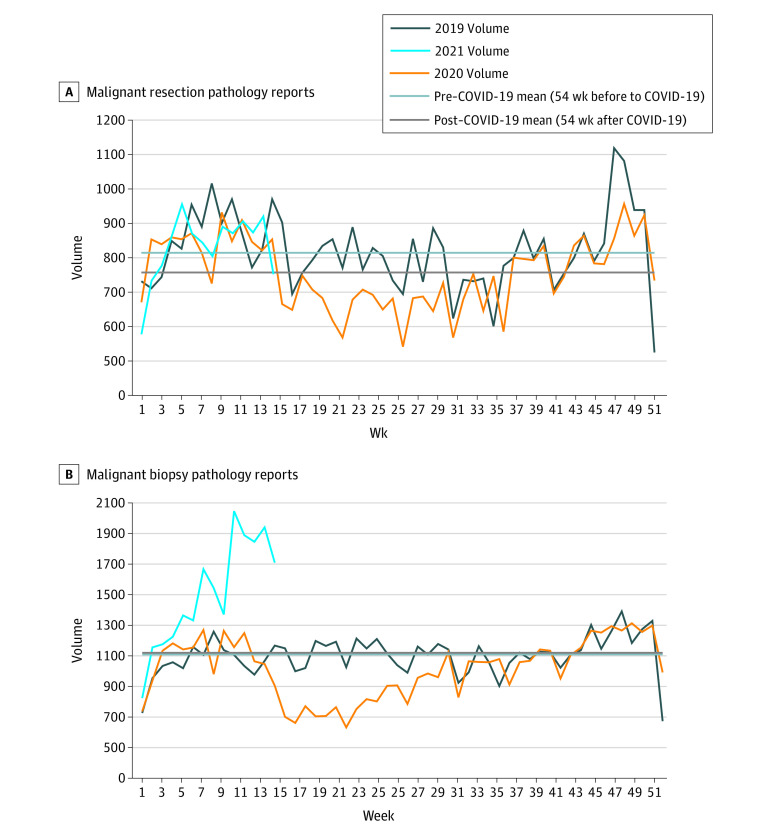
Pathology Report Volumes, Ontario, December 31, 2018, to April 4, 2021

Resections decreased in March 2020 to mid-August 2020 (weeks 14 to 33) (−1.1% to −27.8%), after which the volume began to recover (consistent with the pattern for surgical cancer treatments) (Figure 2). The post–COVID-19 weekly mean was 7.1% lower than the pre–COVID-19 weekly mean.

Biopsies began to decrease by in March 2020 through the end of July 2020 (−11.2% to −41.2%). The volume returned to near prepandemic levels by week 31 and was 68.3% greater than prepandemic levels as of the week of March 29, 2021 (week 14). Because of this surge in 2021, the post–COVID-19 weekly mean was 1.0% higher than the pre–COVID-19 weekly mean.

### Surgical Cancer Treatment 

The volume of P2 to P4 surgical cancer treatments began to decrease in March 2020 after the government’s directive to decrease nonemergent surgical procedures, with the highest reductions in April and May (−34.7% to −37.0%) 2020 (Figure 1). The volume remained 5% to 16% less than prepandemic levels for most months from June 2020 to February 2021. When stratified by priority, a 40.0% reduction was observed for P4 surgical procedures, there was no change to P3 procedures, and the P2 procedure volume was 65.6% higher than in the previous year.

### Systemic Treatment

There were 5500 (−8.2%) fewer new consultations for systemic treatment in the first pandemic year (Table). The greatest reductions were in May to October 2020 (−9.3% to −24.8%) (Figure 1). There were also 17 022 (−3.5%) fewer systemic therapy suite visits (Table). The largest reduction of systemic suite visits was in May 2020 (−14.3%) (Figure 1). The volume fluctuated at approximately prepandemic levels through 2020 before surpassing them in 2021. Changes to systemic visits differed by visit type; changes to parenteral and oral systemic treatment visits were limited, while supportive or adjunctive care visits were reduced by 45% in April and May 2020 and by more than 20% in June to August 2020 (eFigure 3 in Supplement 1). The volume of follow-up visits was 9.3% (66 146) higher in the first year of the pandemic.

New treatment consultations conducted virtually accounted for less than 0.5% of total consultations before February 2020, increased to 10.7% of total consultations in March 2020, and increased further to 28.3% to 49.0% from April 2020 to March 2021 (eFigure 4 in Supplement 1). Follow-up visits conducted virtually increased from 2.1% to 2.6% of total follow-up visits in January 2019 to February 2020 to 46.3% to 54.5% of total follow-up visits in April 2020 to March 2021.

### Radiation Treatment

There were 4783 (−9.3%) fewer new consultations for radiation treatment in the first year of the pandemic (Table). The greatest reductions were in May to October 2020 (−13.3% to −27.2%) (Figure 1). There were also 141 629 fewer (−21.0%) radiation treatment visits (Table), with volumes 10.2% to 32.7% less than prepandemic levels from April 2020 to February 2021. This decrease corresponds to the release of Ontario’s pandemic guidance on increased utilization of hypofractionation. Reductions in treatment visits were consistent by treatment intent.

New treatment consultations conducted virtually accounted for less than 2% of total consultations before February 2020, increased to 12.8% of consultations in March 2020, and increased further to 36.9% to 60.0% of consultations from April 2020 to March 2021 (eFigure 4 in Supplement 1). Virtual follow-up visits increased from 3.3% to 4.6% of total follow-up visits in January 2019 to February 2020 to 49.2% to 69.4% of total follow-up visits in April 2020 to March 2021.

### Psychosocial Oncological Care

There were 310 (1.2%) additional new psychosocial oncological care visits and 6950 more (9.8%) follow-up visits during the first year of the pandemic (Table). The percentage of psychosocial oncological care visits that were virtual also increased beginning in March 2020, with visits conducted virtually ranging from 65.0% to 77.8% of total visits in April 2020 to March 2021 vs 15% to 20% prior to the pandemic (eFigure 4 in Supplement 1).

## Discussion

This cohort study examined changes during the first year of the COVID-19 pandemic in the cancer system in Ontario. While the most clinically urgent services continued, major reductions were observed for most services beginning in March and April 2020, when rigorous hospital surge capacity preservation and infection prevention and control measures were implemented. More than 1.16 million fewer episodes of care were observed during the first pandemic year.

Our findings add to the increasing body of evidence that demonstrates changes to the full spectrum of cancer care globally during the COVID-19 pandemic. Similar reductions were reported across the care continuum in India during the first pandemic wave.^9^ A study of US Medicare beneficiaries also reported major reductions in billings for cancer screening, cancer drugs, biopsies, and surgical treatments.^7^ Some of the greatest reductions were observed in cancer screening, as most jurisdictions suspended services during the first wave.^3^ Reductions in cancer diagnoses similar to those in Ontario have been reported elsewhere.^4,5,7,12,13,14,15,16,17^ In line with our findings, a study from Italy reported a 17% reduction in radiotherapy.^18^ Another study from Spain reported reductions of 37% in new oncology referrals and 38% in new treatments,^19^ substantially greater reductions than those observed in Ontario. In contrast to our findings, which demonstrated that systemic treatment visits were largely unchanged, a national study from Scotland reported a 29% reduction in systemic therapy visits in March 2020.^20^ However, a study from the Canadian province of Manitoba reported little change in parenteral chemotherapy visits,^17^ where similar interim measures were used (eg, shorter regimens). Such differences are expected, given the variability in timing of pandemic waves, policy directives, and clinical guidance.

Our findings demonstrate that while it is possible to decrease service volumes rapidly, recovery can be protracted and complex. While most services had recovered to prepandemic levels 1 year into the pandemic, substantial backlogs accrued which cannot be recovered until volumes exceed baseline levels. Some recovery times are estimated to extend for several years.^21,22,23^ If it is assumed that cancer incidence did not decrease, backlogs in primary care, screening, imaging, and specialist services (primarily surgical procedures) have produced an accumulating backlog of thousands of individuals who may face delays entering and moving through cancer systems.

The COVID-19 pandemic necessitated substantial changes to the delivery of care without time for robust guideline development. Rapid shifts to virtual models of care and changes to personal protective equipment and treatment protocols were critical for risk mitigation and to ensure continuity of care. However, the safety, efficacy, equity, and patient experiences associated with these shifts must be studied. Increasing backlogs also have required rapid development and implementation of management strategies. One strategy is patient prioritization according to disease risk, survival, and quality of life. Prioritization of individuals attending breast cancer screening according to breast cancer risk is estimated to have substantially reduced the mammography backlog for higher risk groups^22^ and shifting low-yield colonoscopy (eg, screening individuals at average colorectal cancer risk) to high-yield (eg, individuals with a positive result on a fecal immunochemical test) is estimated to shorten colonoscopy backlog recovery time.^21^ Initiatives to increase diagnostic imaging and surgical capacity have also been introduced in Ontario, likely associated with the increase in imaging, treatment, and psychosocial oncological care volumes observed in 2021.

A surge of new cancer diagnoses is probable as backlogs are recovered.^24^ We previously estimated that up to 1507 fewer invasive breast cancers, 1222 fewer invasive cervical cancers and cervical precancers, and 462 fewer invasive colorectal cancers would have been detected through Ontario’s organized cancer screening programs in 2020 alone.^25^ In addition to screening, other clinical areas critical to entry into the cancer system, such as primary care and emergency department visits, have been reduced significantly.^26,27^ Of particular concern is whether stage shift will occur associated with diagnostic and treatment delays experienced by the thousands of patients missing from cancer systems during the pandemic. Modeling studies have projected significant impacts on morbidity and mortality.^28,29,30^ This is supported by evidence from systematic reviews. A 2020 review^31^ found that 4-week cancer treatment delay was associated with increased mortality for 7 disease sites. Another 2020 study^32^ found surgical delays exceeding 30 to 40 days for primarily resected colon cancer and time to surgery of longer than 7 to 8 weeks after neoadjuvant therapy for rectal cancer were associated with worse survival outcomes. While we cannot yet measure clinical changes associated with pandemic-related service reductions, anecdotal evidence is emerging from oncologists in Ontario of the presentation of patients with more advanced disease. Ongoing efforts to fully restore cancer services are necessary.

This study has several strengths. In particular, its population-based design, broad coverage of the cancer care continuum, reporting of a full year of pandemic data, and use of high-quality administrative health databases serve to strengthen the quality of evidence.

### Limitations

This study has some limitations. Our design could not account for nonpandemic-related events that occurred during the same period. We were also not able to account for outside factors, such as individual decisions about undergoing care during the pandemic and pandemic-related mortality. Our prepandemic comparison period included the first 2 weeks of the pandemic, which may have led to the underestimation of changes. Overall change was also underestimated because our study did not include some aspects of primary prevention (eg, school-based human papillomavirus vaccinations), cancer-related primary care and emergency department visits, or palliative and end-of-life care.

## Conclusions

The findings of this cohort study suggest profound and unprecedented changes occurred in cancer detection, diagnosis, treatment, and supportive care during the COVID-19 pandemic in Ontario, Canada, and provide evidence of the deficit that has accrued across the continuum of care. Given the integrated nature of this continuum, measurement and recovery efforts must incorporate a systems approach. Additionally, our findings point to the potential for substantial changes in cancer morbidity and mortality. This underscores the urgency of continuing to identify and implement strategies to recover backlogs to restore cancer care and prevent a secondary public health crisis in cancer.

## Notes

Supplement 1. **eTable.** Data Sources **eFigure 1.** Increased or High-Risk Cancer Screening Test Volumes, Ontario, January 1, 2019, to March 31, 2021 **eFigure 2.** Positron Emission Tomography Scan Volumes, Ontario, January 1, 2019, to March 31, 2021 **eFigure 3.** Systemic Treatment Visits by Visit Type, Ontario, January 1, 2019, to March 31, 2021 **eFigure 4.** Volume and Percentage of Treatment and Psychosocial Oncology Visits Conducted Virtually, Ontario, January 1, 2019, to March 31, 2021

Supplement 2.
The Cancer Care Ontario COVID-19 Impact Working Group
