# Supplementary material for: Delivery of Cancer Care in Ontario, Canada, During the First Year of the COVID-19 Pandemic
Source: JAMA Netw Open. 2022 Apr 25;5(4):e228855. doi: 10.1001/jamanetworkopen.2022.8855 (PMC9039771; doi:10.1001/jamanetworkopen.2022.8855)
Supplement: Supplement 1. — eTable. Data Sources eFigure 1. Increased or High-Risk Cancer Screening Test Volumes, Ontario, January 1, 2019, to March 31, 2021 eFigure 2. Positron Emission Tomography Scan Volumes, Ontario, January 1, 2019, to March 31, 2021 eFigure 3. Systemic Treatment Visits by Visit Type, Ontario, January 1, 2019, to March 31, 2021 eFigure 4. Volume and Percentage of Treatment and Psychosocial Oncology Visits Conducted Virtually, Ontario, January 1, 2019, to March 31, 2021 [file jamanetwopen-e228855-s001.pdf]

Supplemental Online Content

Walker MJ, Wang J, Mazuryk J, et al; Cancer Care Ontario COVID-19 Impact Working Group. Delivery of cancer care in Ontario, Canada, during the first year of the COVID-19 pandemic. *JAMA Netw Open*. 2022;5(4):e228855. doi:10.1001/jamanetworkopen.2022.8855

eTable. Data Sources

eFigure 1. Increased or High-Risk Cancer Screening Test Volumes, Ontario, January 1, 2019, to March 31, 2021

eFigure 2. Positron Emission Tomography Scan Volumes, Ontario, January 1, 2019, to March 31, 2021

eFigure 3. Systemic Treatment Visits by Visit Type, Ontario, January 1, 2019, to March 31, 2021

eFigure 4. Volume and Percentage of Treatment and Psychosocial Oncology Visits Conducted Virtually, Ontario, January 1, 2019, to March 31, 2021

This supplemental material has been provided by the authors to give readers additional information about their work.

© 2022 Walker MJ et al. *JAMA Network Open.*

eTable. Data Sources

**Database**	**Volume metrics extracted**
Laboratory Reporting Tool	• Guaiac fecal occult blood tests
FIT Data Submission Portal	• Fecal immunochemical tests
Gastrointestinal Endoscopy Data Submission Portal	• Outpatient colonoscopies performed in hospitals
Ontario Health Insurance Plan Claims History Database	• Outpatient colonoscopies performed at non-hospital clinics<br>• Colposcopies
Cytobase	• Cervical cytology tests
Ontario Breast Screening Program Integrated Client Management System	• Screening mammograms<br>• Breast screening magnetic resonance imaging scans
Wait Time Information System	• Diagnostic/staging magnetic resonance imaging scans<br>• Diagnostic/staging computed tomography scans<br>• Adult cancer treatment surgeries
Ontario Health, Cancer Care Ontario Electronic Mapping, Reporting and Coding Plus	• Malignant resection pathology reports<br>• Malignant biopsy pathology reports[1]
Activity Level Reporting database	• New systemic treatment consultations<br>• All systemic suite visits<br>• Antineoplastic parenteral treatment<br>• Oral antineoplastic treatment visits<br>• Supportive/adjunctive systemic therapy visits<br>• Systemic treatment follow-up visits<br>• New radiation treatment consultations<br>• Radiation treatment visits<br>• Radiation treatment follow-up visits<br>• New psychosocial oncology visits<br>• Follow-up psychosocial oncology visits

1: Including the following biopsy procedures/specimens: bladder curetting, dilation (or dilatation) and curettage, endocervical curetting, prostate chips, punch/shave, transurethral resection of prostate, fine needle aspiration, incisional biopsy, core (needle) biopsy, mammatome biopsy, tru-cut, lymph node biopsy, stereotactic biopsy, polypectomy, excisional biopsy, cone/LEEP/LOOP/LLETZ.

© 2022 Walker MJ et al. *JAMA Network Open.*

eFigure 1. Increased or High-Risk Cancer Screening Test Volumes, Ontario, January 1, 2019, to March 31, 2021

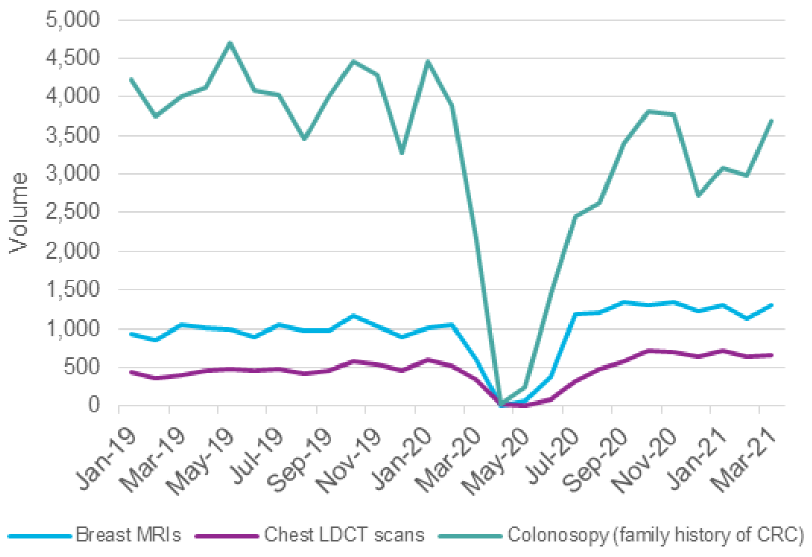

eFigure 2. Positron Emission Tomography Scan Volumes, Ontario, January 1, 2019, to March 31, 2021

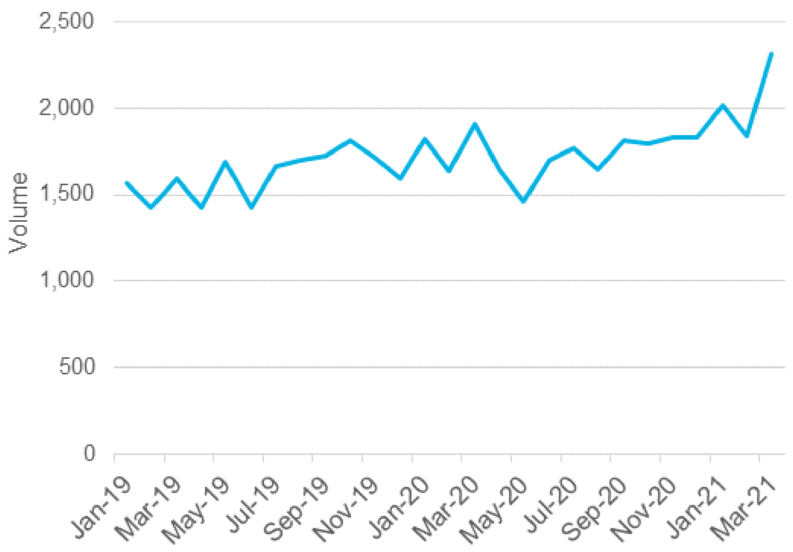

© 2022 Walker MJ et al. *JAMA Network Open.*

eFigure 3. Systemic Treatment Visits by Visit Type, Ontario, January 1, 2019, to March 31, 2021

a) Antineoplastic parenteral treatment visits

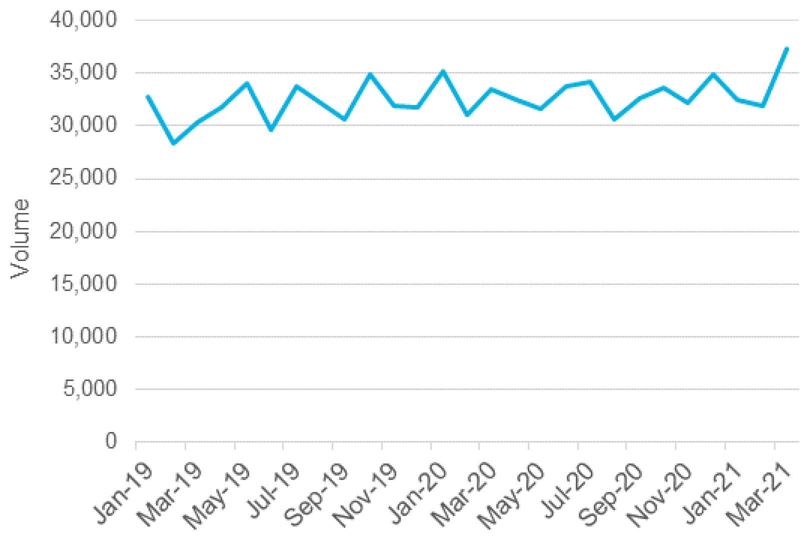

b) Oral systemic treatment visits

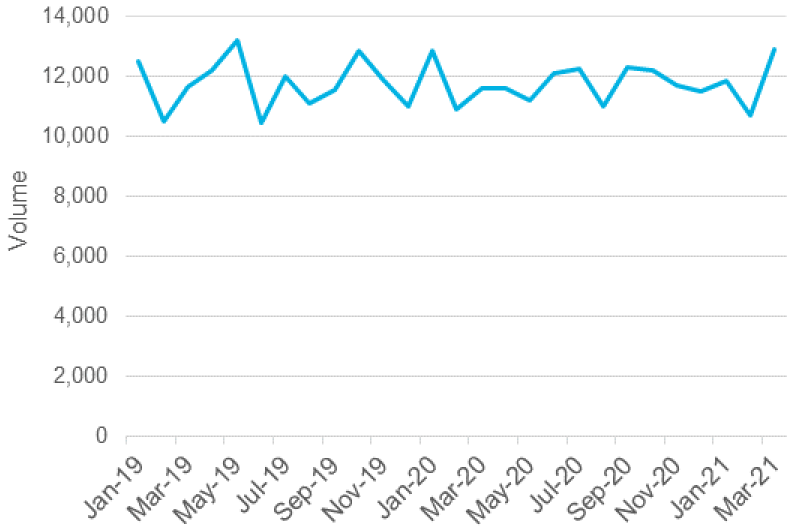

© 2022 Walker MJ et al. *JAMA Network Open.*

eFigure 3. Systemic Treatment Visits by Visit Type, Ontario, January 1, 2019, to March 31, 2021

c) Supportive/adjunctive systemic therapy visits

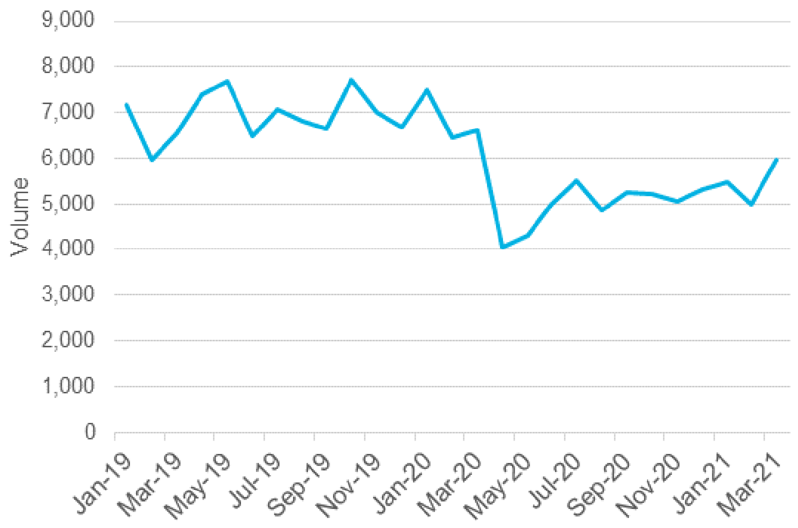

© 2022 Walker MJ et al. *JAMA Network Open.*

eFigure 4. Volume and Percentage of Treatment and Psychosocial Oncology Visits Conducted Virtually, Ontario, January 1, 2019, to March 31, 2021

a) Virtual new systemic treatment consultations

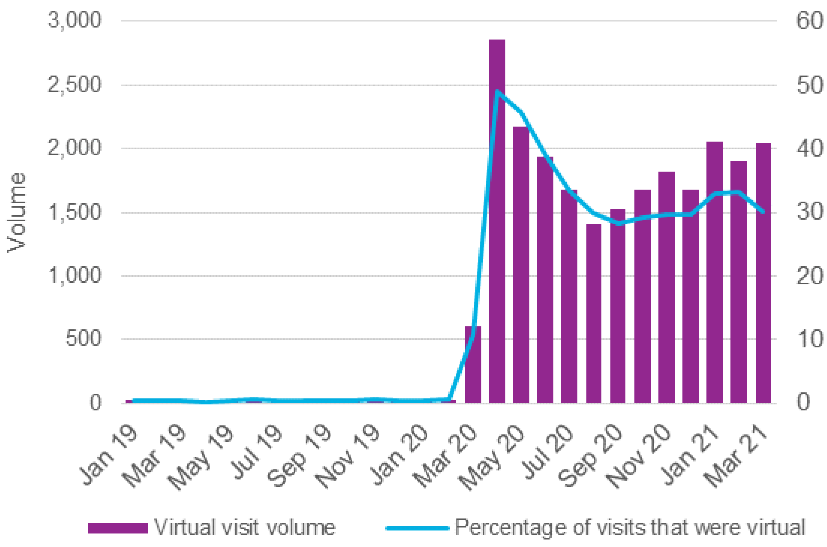

b) Virtual new radiation treatment consultations

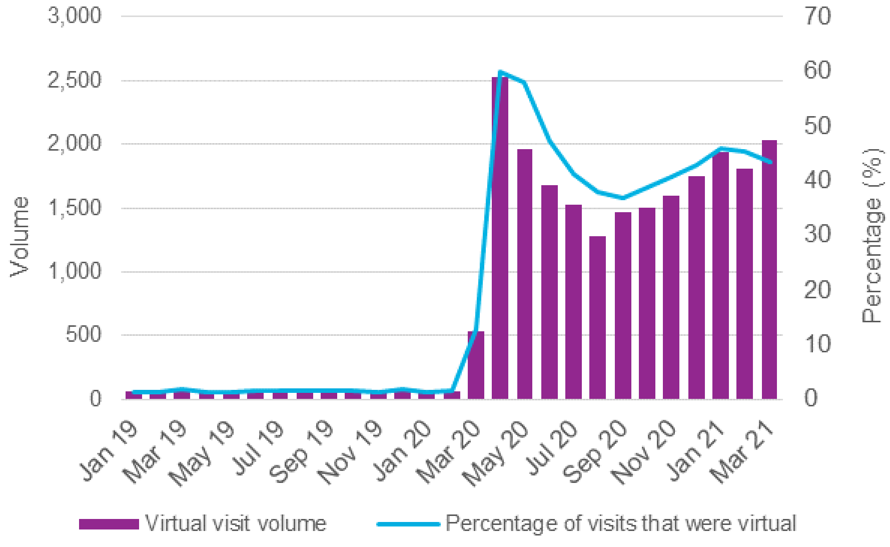

© 2022 Walker MJ et al. *JAMA Network Open.*

eFigure 4. Volume and Percentage of Treatment and Psychosocial Oncology Visits Conducted Virtually, Ontario, January 1, 2019, to March 31, 2021

c) Virtual new psychosocial oncology visits

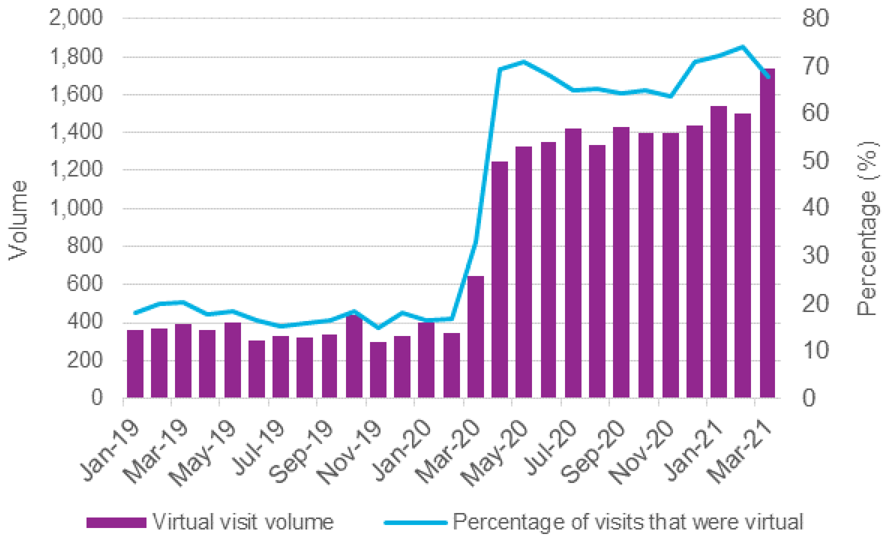

© 2022 Walker MJ et al. *JAMA Network Open.*

eFigure 4. Volume and Percentage of Treatment and Psychosocial Oncology Visits Conducted Virtually, Ontario, January 1, 2019, to March 31, 2021

d) Virtual systemic treatment follow-up visits

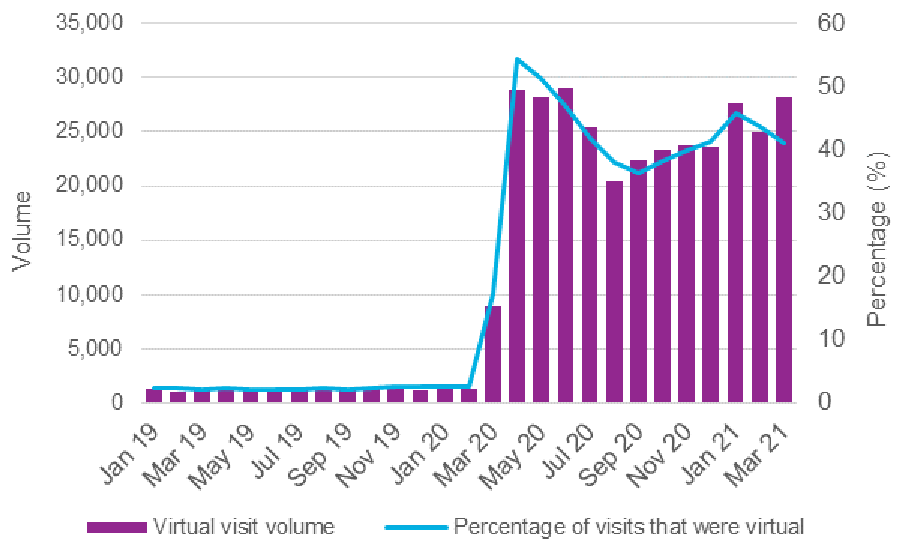

e) Virtual radiation treatment follow-up visits

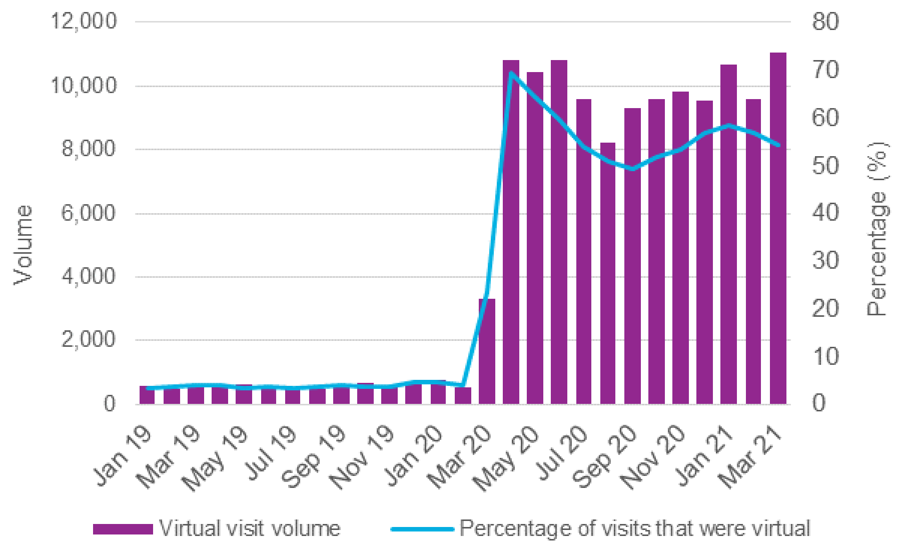

© 2022 Walker MJ et al. *JAMA Network Open.*

eFigure 4. Volume and Percentage of Treatment and Psychosocial Oncology Visits Conducted Virtually, Ontario, January 1, 2019, to March 31, 2021

f) Virtual psychosocial oncology follow-up visits

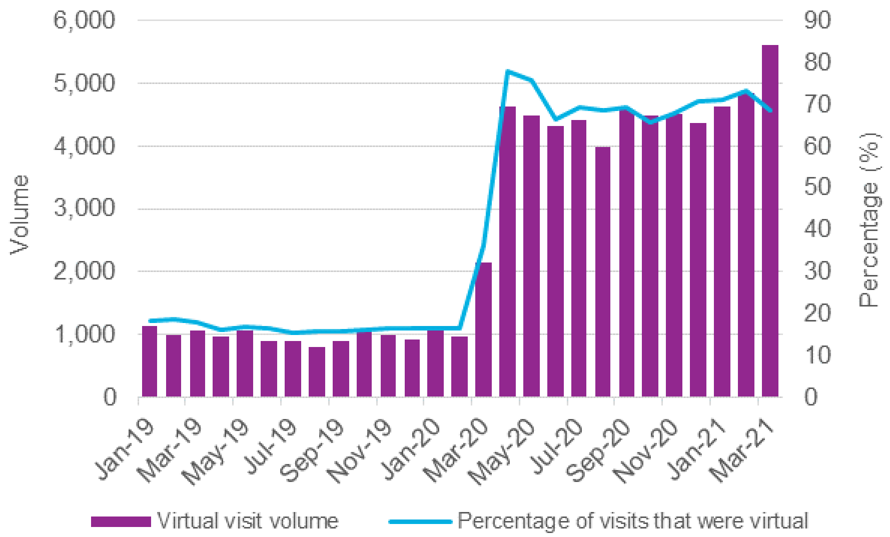

© 2022 Walker MJ et al. *JAMA Network Open.*
