# Supplementary material for: Delivery of Cancer Care in Ontario, Canada, During the First Year of the COVID-19 Pandemic
Source: JAMA Netw Open. 2022 Apr 25;5(4):e228855. doi: 10.1001/jamanetworkopen.2022.8855 (PMC9039771; doi:10.1001/jamanetworkopen.2022.8855)
Supplement: Supplement 2. — The Cancer Care Ontario COVID-19 Impact Working Group [file jamanetwopen-e228855-s002.pdf]

Supplemental Online Content: Nonauthor Collaborators

*Indicates required information. Only first name, last name, and suffix will appear in PubMed.

*Group Name(s): Cancer Care Ontario COVID-19 Impact Working Group							
***First Name and Middle Initial(s)**	***Last Name**	***Suffix (eg, Jr, III)**	Academic Degrees	Institution	Location (city, state/province, country)	Role or Contribution, eg, chair, principal investigator	Group (if more than 1 Group listed in the byline) and/or Subgroup (eg, Steering Committee)
Chamila	Adhihetty						
Jaclyn	Beca						
Diane	Burns						
Catherine	Chan						
Kelvin KW	Chan						
Lauren	Chun						
Melissa	Coulson						
Gail E	Darling						
Prithwish	De						
Steven	Dong						
Catherine	Dubé						
Maria	Eberg						
Samantha	Fienberg						
Colleen	Fox						
Sophie	Foxcroft						
Ron	Fung						
Scott	Gavura						
Natasha	Gray						
Sherrie	Hertz						
Brian	Ho						
Claire M	Holloway						
Amber	Hunter						
Nathaniel	Jembere						
Tina	Karapetyan						
Shivali	Kaushal						
Mary J	King						
Ordeena	Linton						
Aisha K	Lofters						
Andrea	Mackesy						

Supplemental Online Content: Nonauthor Collaborators

*Indicates required information. Only first name, last name, and suffix will appear in PubMed.

***First Name and Middle Initial(s)**	***Last Name**	***Suffix (eg, Jr, III)**	Academic Degrees	Institution	Location (city, state/province, country)	Role or Contribution, eg, chair, principal investigator	Group (if more than 1 Group listed in the byline) and/or Subgroup (eg, Steering Committee)
Faisal	Majeed						
Jessica	Mann						
Pamela	MacCrostie						
Garth	Matheson						
Bronwen R	McCurdy						
Elaine	Meertens						
Reeza	Menalo						
Lisa	Milgram						
Nicole	Moleschi						
Elena	Mow						
K Joan	Murphy						
Rohini	Naipaul						
Karen	Nguyen						
Diane	O'Grady						
Aaron	Pollett						
Elizabeth	Quilliam						
Dimpho	Radebe						
Jocelyn E	Sacco						
Nancy	Shukla						
Vicky	Simanovski						
Steve	Scott						
Xiaochen	Tai						
Cristina	Tassone						
Arany	Theivendram						
Rebecca	Truscott						
Christa	Wang						
Audrey	Wong						
Lyndee	Yeung						
Marta	Yurcan						
